# Integrity in Business

**Published:** 1872-12

**Authors:** 


### INTEGRITY IN BUSINESS.

### HONEY.

An experienced correspondent of *The Farmer's Home Journal* advises that honey should be put up in glass cans; he might well have said that it is not safe to use any other kind for the preservation of fruits and vegetables. It is best to put in a nice white piece of honeycomb first, and pour the pure honey upon it; this convinces the purchaser that it is the real article.

The honey should be first strained, then warmed and properly sealed. Quart cans are the most convenient size, and are packed in saw-dust, in a box, selling at thirteen dollars a dozen, with a demand very greatly in advance of the supply, because, in the course of a few years, every purchaser becomes a living advertisement, the article being always pure and good, and of full weight and measure.

There are two points here which merit the attention of both citizens and farmers. This correspondent's brand is known to be always good. He can sell more than he can supply, and at his own price.

### PURE MILK.

New Yorkers pay ten cents a quart for their milk; the farmer delivers it at the railroad station for three, one cent for railroad freight, and the remainder of the ten cents goes into the pocket of middle men, those who purchase from the farmer, to sell to the citizen; many of these middle men adding a quart of water or more to each gallon of milk. If it was swill milk, the more water the better. It seems a great shame that the farmer does not get more for his milk, but it is his own fault; any dozen or twenty of them could combine, appoint one of their number to sell in New York, direct to his own customers, the pure article with all its cream. It would not be long before he could sell more than he could procure. Mr. Canfield acted on this principle some years ago, and has made the plan handsomely profitable.

The same thing might be done in reference to other kinds of provisions of large consumption. A man could make a fortune by keeping an apple store or a potato store, sorting them out as Nos. 1, 2, 3, 4, selling them at corresponding prices; letting it be understood that every apple in No. 1 is of the best; and each layer down to the bottom being as good as the first one visible, with prices corresponding to the number. In a very short time two results would be inevitable; he would be able to sell more than any other man, and at higher prices.

It was precisely on this principle that A. T. Stewart laid the foundation-stones of his colossal business, in his little one-room store on Broadway, many years ago. It was soon found out that when he sold an article of dry goods as the best article in the market, it was always the best; not generally so, but infallibly the best. If a second or third rate article was wanted, he had it on hand, but sold it as such, and at a correspondingly lower price. Thus he was able to fix his own prices, and get them too. The people will find their way to the places where they get what they purchase, always, and pay a good price willingly, counting their well-placed confidence as worth something.

### DRESSMAKING.

Much has been said about the destitution of sewing women, and the miserable pittance they get for their work. Yet every mother with a family of daughters knows the great difficulty of getting a competent, honest, and discreet dressmaker. Those known to be such must be engaged weeks, and even months beforehand, at two or three dollars a day, and found.

There are places with liberal pay in every department of business, waiting for competent, faithful persons to fill them. There are many persons all the time on the verge of destitution in any great city, and always in New York; but it will be uniformly found that they have been unfaithful or incompetent, generally both. Persons who engage themselves on a salary, seem to make it a study to do the least possible and yet be able to keep their places. The fewest number of workers will do a thing as well for another as for themselves, even when employed at their own price; they get to the very last cent what was agreed to be given, and the withholding of a single dime on a job of fifty dollars, would excite indignant anger; but let such a person be asked to reply in all sincerity and conscientiousness, "Did you really perform that work as well as you could?" Not one in a multitude could fearlessly answer, "I did."

Printers are among the more intelligent classes, and yet none know so well as they the multitudes of little ways in which gross cheateries can be perpetrated, and are too often practised.

Has the reader never purchased a newspaper, and, on opening it, found it was of such a flimsy material as to almost fall to pieces, unless the greatest care possible were used in the handling? It is because it was not honest paper; ten, twenty or more per cent. was lime, purchasable for half a cent a pound, the paper costing ten cents or more a pound. In this case, the paper maker and the publisher divide the profits the people pay them.

You send an advertisement to a second or third rate establishment, charging so much a line. One word over is considered a line, even if it contain but two letters to make it reach a word over. There is a process of spreading the words of the previous line, giving much wider spaces between the words than is at all necessary. If the man setting the type knew that he was to pay by the line, he could very easily save it.

Then again the indifference and carelessness of workmen is amazing. Look at carpenters! how they waste nails ; how they cut up timber to a disadvantage ; how carelessly they measure! In old times it was a proverb, " Measure twice, and cut once." All take that trouble when the boards are their own ; the fewest number when they belong to other people.

### WHITE PAINT.

A few years age it was essential in some parts of machinery to have a pure article of white lead ; no intelligent workman ever thought for a moment of purchasing white lead made in the United States ; perhaps it is the same now, because not a pure pound could be had nearer than England ; so the knowing ones always stipulated that their houses should be painted with English white lead, which always sold at a higher price than American-made. Whenever you see a building painted white on the outside, with a darkish color in many places, you may be sure that it is American white lead, selling, perhaps, at ten cents a pound, but adulterated ten, fifty, eighty per cent. with barytes, which costs less than a cent a pound. Ask the maker of the article about it, and he will tell you, without any hesitation, that barytes improves the white lead ; but ask him three questions : —

1st. How does barytes improve the quality of white lead ?

2d. If a little improves it, more would make it better? why not then make your white paint out of barytes altogether ?

3d. If pure white lead is worth ten cents a pound, and barytes but one cent, and is just as good, what is the use of making white lead at all ?

Examine the professions. The lawyer too often encourages his client to bring suit or defend a suit, when, if it had been his own, he would have compromised. And

THE DOCTOR, that is, the "irregular," the number tens among them, will, in a large majority of cases, assume a knowing look, and tell his trusting applicant he will certainly cure him; he receives his fee, and the patient his physic. Well, he doctors on for awhile, and there being no perceptible change except the weight of the purse, he finds that a new symptom just developed makes it necessary to go to the South.

Two gentlemen not long ago were offered a very valuable property; but not being willing to trust their own judgment, they called upon their mutual friend Mr. L., No. — Fifth Avenue, stated the object of their visit, and the full particulars. Mr. L. said it required some reflection, as it involved a large amount of money; but if they would call at twelve next day, at his office, he would give his views. Next morning, before breakfast, he walked around to see the property, and was very much pleased with it; in fact, so much so, that he purchased it himself, and cleared, by that

"OPERATION,"

sixty-one thousand dollars.

We are not worse in morals in our day and generation, than in the time of our fathers; but we are, however, not so good but that there is room for improvement, and the foundation must be laid on the Bible, and by the mothers of the land. They must by their holy teachings, begun as soon as the infant lips can whisper "Our Father who art in heaven," impress on the young heart in earnestness of spirit, to be repeated every day, that there is an all-seeing eye, and that all wrong-doing makes the angels weep.

Meanwhile, let every young man now entering upon the business of life, remember that in every country and in every clime the surest road to permanent success in life is

HONEST DEALING, bringing with it, uniformly, a quiet, peaceful, and happy old age.

